# Heteroplasmy Detection of Mitochondrial DNA A3243G Mutation Using Quantitative Real-Time PCR Assay Based on TaqMan-MGB Probes

**DOI:** 10.1155/2018/1286480

**Published:** 2018-11-13

**Authors:** Enguang Rong, Hanbo Wang, Shujing Hao, Yuhong Fu, Yanyan Ma, Tianze Wang

**Affiliations:** ^1^Chinovo Laboratory, Beijing, China; ^2^Beijing Dong Cheng District Community Health Service Management Center, Beijing, China; ^3^Department of Pediatrics, Affiliated Hospital of Qinghai University, Xining, China; ^4^Clinical Laboratory of Chinovo, Beijing, China

## Abstract

A point mutation of mitochondrial DNA (mtDNA) at nucleotide position 3243 A to G (mt.3243A>G) is involved in many common diseases, including maternally inherited diabetes and deafness (MIDD) and mitochondrial encephalomyopathy, lactic acidosis with stroke-like episodes (MELAS). However, the mutant level of mt.3243A>G varies both among individuals and in different organs, tissues, and even cells of single individuals. For detection of this mutation, current methods have limited universality and sensitivity and may be not adequate for a routine clinical test. Here, we develop and evaluate a rapid TaqMan-MGB quantitative real-time PCR (qPCR) method for detecting and quantifying the heteroplasmy level of mt.3243A>G in single-tube analysis. With our method, the sensitivity of detection was as low as 0.1%, but the accuracy of quantification was reliable, down to 4%. All positives could be correctly identified, and the heteroplasmy levels determined by qPCR correlated well with the results from restriction fragment length polymorphism (RFLP) and pyrosequencing assays (r = 0.921~0.973 and 0.972~0.984). In addition, we demonstrated that the urinary sediments, leukocytes, or hair follicles might be ideal templates to detect and quantify the heteroplasmy of mt.3243A>G mutation; however, they should be optimized or retreated for further accurate quantification. Our study should allow rapid and high throughput diagnostic testing and can potentially be used to clarify the association between clinical phenotype and pathogenic mitochondrial mutations derived from various tissues.

## 1. Introduction

Mitochondria are key organelles that have an essential role in the life and death of cells [1]. Inherited mitochondrial DNA (mtDNA) mutations have been responsible for a variety of neuromuscular disorders. For a clinically proven mutation to manifest as a diseased phenotype, as in the case of primary mitochondrial disorders, the allele frequency (heteroplasmy) needs to exceed a certain threshold, referred to as the phenotypical threshold effect [2]. The mtDNA A3243G (mt.3243A>G) mutation in the tRNA^Leu(UUR)^ gene is one of the most common mtDNA point mutations [3]. This mutation is associated with a wide range of clinical manifestations including mitochondrial encephalomyopathy, lactic acidosis with stroke-like episodes (MELAS) [4, 5], and maternally inherited diabetes and deafness (MIDD) [6, 7]. However, the phenotypic expression of the mt.3243A>G can be highly variable from tissue to tissue, making mutation analysis important for diagnosis and management [8].

The determination of the mt.3243A>G heteroplasmy level contributes to investigating relevance to clinical presentations, sensitivity of genetic diagnosis, and genetic counseling for disease transmission. However, the level of mt.3243A>G varies both among individuals and in different organs and tissues of single individuals; it is thought that the load of mutant is in part responsible for the varied clinical expression of mtDNA defects in general [1, 8]. Although several studies have found no correlation or only a weak correlation between mutation load in blood and clinical phenotype, there is some correlation in degree of mt.3243A>G heteroplasmy with the age of diabetes onset and severity of deafness [9–11]. In contrast, a good correlation between frequency of typical clinical features and heteroplasmy level of mt.3243A>G is seen in muscle, brain, or endocrine organs [12, 13]. Unfortunately, these tissues are not easily accessible for diagnostic testing. Until now, the heteroplasmy level of mt.3243A>G mutation is still often predicted from blood samples in clinical research [14, 15]. More studies are required using accurate methods of heteroplasmy quantification from various tissues as an individual basis.

Current methods for detecting mt.3243A>G include densitometry of ethidium bromide-stained PCR products, Southern blotting of restriction digestion fragments, denaturing high-performance liquid chromatography, and pyrosequencing, which are relatively simple but insensitive [16–20], while the radiolabeled PCR-restriction fragment length polymorphism (PCR-RFLP) assay is sensitive but radioactive [18]. Additionally, ligation-mediated PCR, amplification refractory mutation system (ARMS), or peptide nucleic acid binding assay is overly sensitive and time-consuming [21–23].

Here, we introduce a TaqMan-MGB quantitative real-time PCR (qPCR) approach for accurately quantifying the heteroplasmy level of mt.3243A>G mutation. This is a rapid, sensitive, and accurate technique that allows high throughput and multi-template diagnostic testing. Our study can be applied to any diagnostical samples without the need of post-PCR manipulation.

## 2. Materials and Methods

### 2.1. Patients and Controls

A total of 13 Chinese patients with a diagnosis of MELAS or mitochondrial diabetes (9 males and 4 females, 8-62 years old) were recruited from the outpatient clinic of the Affiliated Hospital of Qinghai University (Xining, China). All patients gave written informed consent before participation in this study, which was approved by the Ethics Committee of the Affiliated Hospital of Qinghai University. Healthy adult volunteers also were recruited after giving informed consent. Peripheral blood, saliva, random urine, and hair follicles were collected from all patients or controls. Total nucleic DNA (nDNA) was extracted from peripheral blood cells or urinary sediments using a standard phenol-chloroform procedure [24] and stored at -20°C for genotyping. The exaction of mitochondrial DNA (mtDNA) was applied as previously described [25]. Leukocytes and mitochondria, or supernatants and precipitations related to isolated mitochondria, were also isolated from peripheral blood [26, 27].

### 2.2. Genotyping

Wild-type (A) and mutant (G) sequences at nucleotide 3243 of the mitochondrial genome were identified by quantitative real-time PCR (qPCR) assay. Mutation discrimination was achieved in single-tube analysis by the use of probes that differed only at the position of the mutation (Table 1). Probes were labeled at the 5′ end with either VIC or 6-FAM and at the 3′ end with a minor groove binding (MGB) protein. The method was validated by mixing experiments and standard curve analysis. Wild-type and mutant plasmids were mixed to generate standard samples with the mt.3243G (mutant) allele at concentrations ranging from 0 to 100%, which were used to determine the minimum detection limit using a standard curve.

PCR was repeated for 40 cycles of denaturation for 5 seconds at 94°C and annealing/extension for 30 seconds at 60°C. The reaction mixture contained 5 *μ*L of* TransStart*® Probe qPCR SuperMix (TransGen, Beijing, China), 0.2 *μ*mol/L of each primer, 0.2 *μ*mol/L of wild-type- and mutant-specific probe, 1× Passive Reference Dye, and 1 *μ*L of templates in a total volume of 10 *μ*L. The templates could be nDNA, mtDNA, leukocytes, urinary sediments, hair follicles, and so on. The fluorescent signal intensities were recorded and analyzed by an ABI QuantStudio™ 6 Flex system (Thermo Fisher, Carlsbad, CA) using the QuantStudio 6 (version 1.1) software. The total time was 1 hour and 15 minutes (55 minutes for loading of samples to the result analysis and 20 minutes for data analysis). For nonisolated DNA samples, conventional PCR amplication was first performed and subsequently purified the products using the* EasyPure*® PCR Purification Kit (TransGen, Beijing, China).

### 2.3. PCR-Restriction Fragment Length Polymorphism (RFLP)

All positives with mt.3243A>G mutation were analyzed by RFLP [21]. Simply, the PCR products were digested with* Apa*I, and a total of 10*μ*l reaction mixture contains CutSmart buffer 1*μ*l (NEB Biolabs, America), PCR product 5*μ*l, and* Apa* I 0.25*μ*l (NEB Biolabs, America), reacted at 25°C for 2 hours after mixing, and then stained with ethidium bromide after agarose gel electrophoresis. The gray values were used to calculate the heteroplasmy of mt.3243A>G.

### 2.4. Pyrosequencing Assay

All positives with mt.3243A>G mutation were analyzed by pyrosequencing assays. The reaction mixture contained 5 *μ*L of PCR buffer (TransGen, Beijing, China), 0.2 *μ*mol/L of each RFLP primer, and 1 *μ*L of templates in a total volume of 10 *μ*L. PCR was denatured for 2 minutes at 94°C and this was repeated for 35 cycles of denaturation for 5 seconds at 94°C, annealing for 30 seconds at 52°C and extension for 30 seconds at 72°C, then extension for 5 minutes at 72°C, and then kept at 4°C. The PCR products were stained with ethidium bromide after agarose gel electrophoresis. The peak height G/A ratios was used to calculate the heteroplasmy of mt.3243A>G.

### 2.5. Statistical Analysis

Mixing experiments and standard curve analysis were used to validate the approach as described previously [28]. The minimum detection limit was determined by correlation analysis,* t*-test, and regression analysis. Quantification of wild-type and mutant mtDNA was calculated by comparing the difference between wild-type and mutant threshold cycles (ΔΔCt). The results were then normalized to a known mixture of 50% heteroplasmy. The ratio of mutant to wild-type DNA was done using the equation 2^−ΔΔCt^ assuming the amplification efficiencies be the same for wild-type and mutant and close to 100% [29–31]. Samples were analyzed in triplicate. All collected data were analyzed by SPSS 17.0 software package. Data were presented as mean and standard deviation (S.D.).* P *values <0.05 were considered statistically significant, and* P* values <0.01 were considered highly significant.

## 3. Results

### 3.1. Evaluation of the TaqMan-MGB qPCR Assay

**Figure 1:**
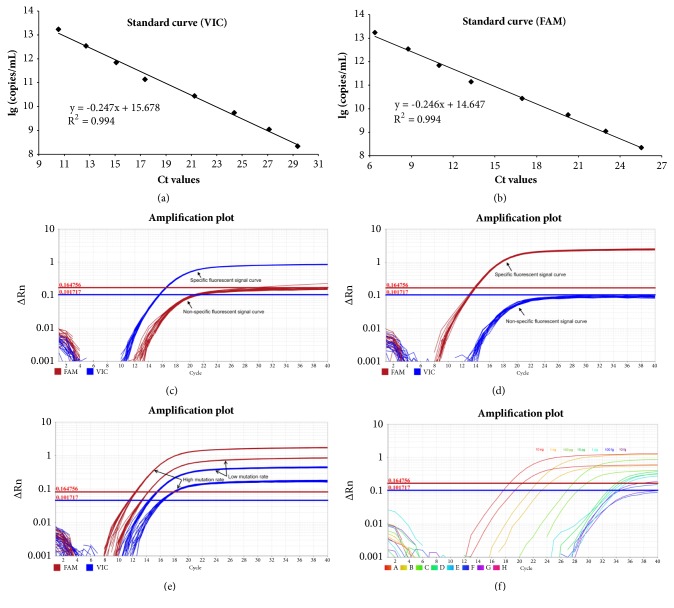
**Standard curves and real-time fluorescence PCR amplification curves. **(a-b) Standard curves of wild-type (VIC, a) and mutant (FAM, b) probes. The standard samples (heteroplasmy level, 50%) were successively diluted five-fold to eight points. All samples were performed in triplicate. (c-d) Amplification curves of 100% WTP (c) and 100% MutP (d) templates. All samples were performed thirty times. (e) Amplification curves of low and high mutation rate templates under exposure to visible light with 30, 45, and 60 minutes. All samples were performed in triplicate. (f) Amplification curves of mtDNA templates with the concentration of 10 fg to 10 ng.

The approach was validated and calculated by mixing experiments and standard curve analysis. To establish the standard curves, we first constructed the wild-type and mutant plasmids (WTP and MutP) with the mt.3243A and mt.3243G target, respectively. WTP and MutP were equally mixed to generate the standard samples. The standard curve analysis indicated the linear relationship between Ct (cycle threshold) and WTP/MutP copies (R^2^ = 0.994). In addition, standard curves revealed that the detection of wild-type and the detection of mutant alleles were equally efficient, with curve gradients of -0.247 and -0.246, respectively (Figures 1(a) and 1(b)). We then investigated the precision of TaqMan-MGB qPCR assay [28]. As expected, either VIC (blue curves) or FAM (red curves) probe was consistently amplified in the corresponding system within 100% WTP or 100% MutP. However, both VIC and FAM probes exhibited nonspecific amplifications. Hence, proper placement of analysis threshold is nevertheless very important. In this study, we determined the fluorescence threshold of VIC and FAM probes by manual analysis at the levels of 0.101717 and 0.164756, respectively (Figures 1(c) and 1(d)). The within-run coefficient of variation (CV_W_) is 3.89% and 4.12%, and the population coefficient of variation (CV_p_) is 7.36% and 7.80%, for the samples with the 3243 mutant alleles present at levels of 30% and 70%. Furthermore, it is negligible for the experimental impact of visible light within 60 minutes, because of the acceptable CV_W_ (5.49% and 4.74%) for the templates of low and high mutation rate (Figure 1(e)). We also found that both mtDNA and nDNA are ideal templates. Take the accuracy of quantification into consideration, the recommended nDNA dosage is 100 pg to 1 *μ*g (data not shown), and the recommended mtDNA dosage is 10 pg to 100 ng per 10 *μ*L PCR reaction mixture (Figure 1(f)). These observations indicated that TaqMan-MGB qPCR assay might be an ingenious, convenient, and accurate method to quantify the heteroplasmy of mt.3243A>G.

### 3.2. Determination of the Minimum Detection Limit

**Figure 2:**
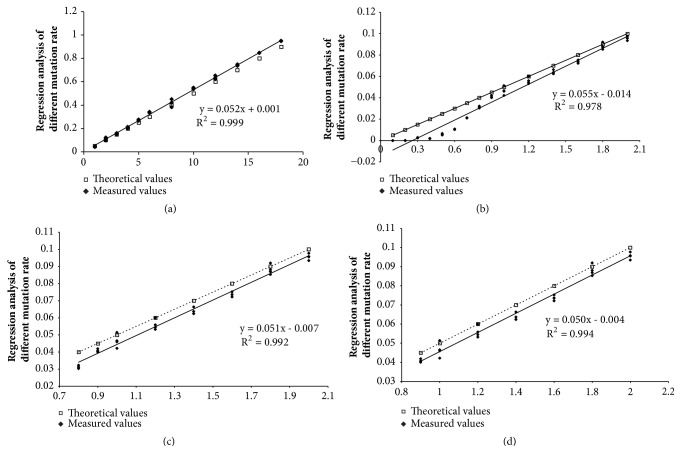
**Regression analysis of the theoretical and measured heteroplasmy values of the standard samples with different mutation rates. **(a–d) Regression analysis of the two datasets derived from the standard samples with the mutation rates of 5 to 90% (a), 0.1 to 10% (b), 4 to 10% (c), and 4.5 to 10% (d). All samples were performed in triplicate.

To determine the detection limit of TaqMan-MGB qPCR assay, we prepared the standard samples with different heteroplasmy levels. By generating serial dilutions of the 100% MutP with 100% WTP to generate samples with the 3243 mutant alleles present at levels of 90, 80, 70, 60, 50, 40, 30, 25, 20, 15, 10, and 5%. The sample with a concentration of 5% mutant mtDNA could be accurately detected by this method (data not shown). There was no difference between the theoretical and measured heteroplasmy values by* t*-test, and the correlation coefficient was 0.9996. Regression analysis also exhibited that the appropriateness degree was good between the two datasets (R^2^ = 0.999, Figure 2(a)). Therefore, we failed to ascertain the minimum detection limit until this step. We then mixed new standard samples with lower heteroplasmy levels of 10, 9, 8, 7, 6, 5, 4.5, 4, 3.5, 3, 2.5, 2, 1.5, 1, 0.5, and 0.1%. Expectedly, there was significant difference between the theoretical and measured heteroplasmy values by the* t*-test (*P*<0.05) in spite of the strong positive correlation (r = 0.9890). In addition, the appropriateness degree was relatively disappointing by regression analysis (Figure 2(b)). We subsequently deleted the measured values of lower heteroplasmy samples and reanalyzed. Finally, we determined the minimum detection limit, which was 4 to 4.5% (Figures 2(c) and 2(d)). The extent of cross-hybridization was analyzed by the mutant probe from a pure plasmid sample. Low levels of cross-hybridization are a common and aberrant observation where the reaction mixture is with low positives. A concentration of mutant mtDNA in samples as low as 0.1% was detected by this method; however, the accuracy of quantification is reliable, down to 4%. Thus, our data shows that TaqMan-MGB qPCR assay is a point device to screen a large number of samples for mt.3243A>G mutation, whose heteroplasmy is higher than 4%.

### 3.3. Quantification of the Positive Samples

**Figure 3:**
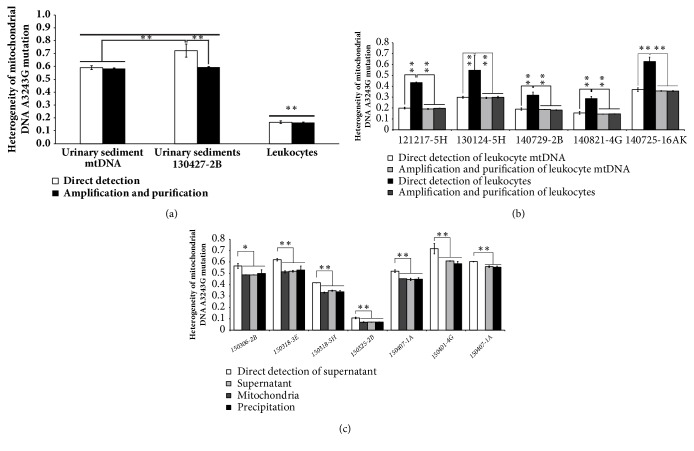
**Heteroplasmy levels of mt.3243A>G mutation by TaqMan-MGB qPCR assay from positive mtDNA and nonisolated DNA samples. **(a–c) Heteroplasmy levels of positives from mtDNAs (a, b), urinary sediments (a), leukocytes (a, b), mitochondria (c), or supernatants and precipitations related to isolated mitochondria (c). All samples were performed in triplicate. The data of each identical sample are expressed as means ± S.D., which are derived from triplicate determinations. ^*∗*^*P*<0.05; ^*∗∗*^*P*<0.01.

**Figure 4:**
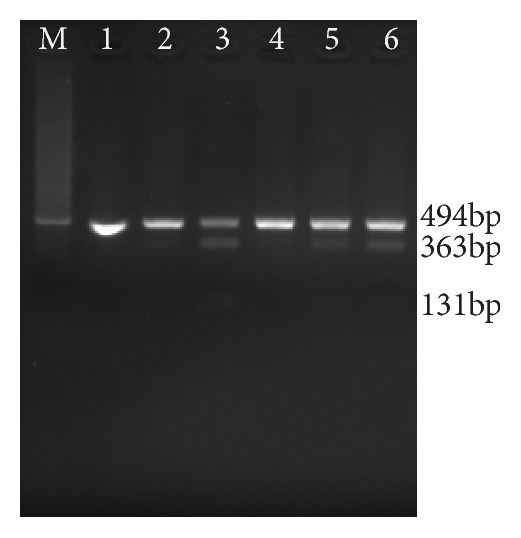
**PCR-RFLP assay of mtDNA A3243G positive samples. **M: marker (500bp); 1-6: the 6 positive samples (1-140821-4G; 2-121217-5H; 3-130427-2B; 4-140729-2B; 5-130124-5H; 6-170725-16AK).

**Figure 5:**
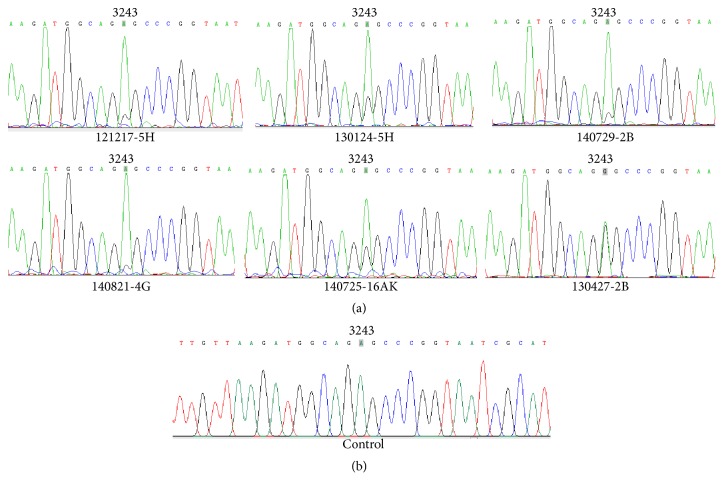
Pyrosequencing assay of mtDNA A3243G positive (a) and control (b) samples.

We have 6 positive mtDNA samples. One of them was isolated from urinary sediments, and the others came from leukocytes. Meanwhile, we have 27 negative nDNA and 8 negative mtDNA samples, all of which were exacted from leukocytes. All 6 known positives were identified by the TaqMan-MGB qPCR method. None of the known negatives were scored as positive (Table 2). We used the crossing point data to calculate the percentage of heteroplasmy of the positive samples. In addition, we quantified them by PCR-RFLP and pyrosequencing technology. Levels of heteroplasmy ranged from 14 to 60% (Figures 3(a) and 3(b)), as determined by our method, and compared with PCR-RFLP (Figure 4) and pyrosequencing (Figure 5) results (r = 0.973 and 0.984, respectively).

To simplify the experimental duration, we selected the nonisolated DNA samples as the templates, such as EDTA-anticoagulated blood, isolated leukocytes, salivary precipitations, urinary sediments, and hair follicles. Interestingly, we found that leukocytes, urinary sediments, and hair follicles were all ideal templates to amplify directly and quantify. In this case, we identified the 6 known positives and calculated their heteroplasmy again. As shown in Figures 3(a) and 3(b), the heteroplasmy could be detected by TaqMan-MGB qPCR method from urinary sediments and leukocytes. For the same person, level of heteroplasmy in urinary sediments was higher than that in leukocytes by 3.5-fold (*P*<0.01, Figure 3(a)). These data suggested that the heteroplasmy levels of mt.3243A>G could be directly quantified by quantitative PCR from DNA samples, and even from urinary sediments, leukocytes or hair follicles.

### 3.4. Feasibility of Nonisolated DNA Samples as the Templates

Although the heteroplasmy of mt.3243A>G can be indentified from the crude urinary sediments and leukocytes, the mutation levels are generally higher than those from the mtDNAs (*P*<0.01, Figures 3(a) and 3(b)). Is it just a coincidence that we select the nonuniform urinary sediments or leukocytes, where it happened to include the cells with higher heteroplasmy of mt.3243A>G, or some unknown substances in it disturb the binding of FAM and VIC probes? To validate this hypothesis, we first performed conventional PCR amplication using the urinary sediments or leukocytes as the templates, and subsequently purified the products to develop qPCR assay. Interestingly, the amplified and purified products, from mtDNAs, urinary sediments, or leukocytes, exhibited similar heteroplasmy levels of mt.3243A>G to original mtDNA samples (Figures 3(a) and 3(b)). That is to say, there was something unknown, which might be enzymes or solid components, that interferes with the accuracy of mt.3243A>G heteroplasmy level. To further demonstrate the conclusion, we searched for another 7 positive samples, which contained mitochondria, supernatants, and precipitations related to isolated mitochondria. Apart from supernatants, neither mitochondria nor precipitations efficiently quantified the heteroplasmy of mt.3243A>G mutation (Figure 3(c); data not shown). However, after amplication and purification, the heteroplasmy levels of mt.3243A>G were close to each other among the above three types of samples and were all significantly lower than those from direct templates of supernatants (Figure 3(c)). Levels of heteroplasmy of the 7 positives ranged from 7 to 60% (Figure 3(c)), as determined by our method, and were compared with PCR-RFLP and pyrosequencing results (r = 0.921 and 0.972, resp.). Taken together, we clarified that the urinary sediments, leukocytes, or hair follicles were not suitable templates to precisely quantify the heteroplasmy of mt.3243A>G mutation; if necessary, they should be optimized or retreated before quantificaiton.

## 4. Discussion

In the present study, we have exhibited a special quantitative real-time PCR assay for simultaneous detection and quantification of mt.3243A>G mutation heteroplasmy (Figure 1). This TaqMan-MGB qPCR method is easy and rapid in providing accurate results down to a level of 0.1% mutant mitochondria; however, the accuracy of quantification is reliable, down to 4% (Figure 2), which seems to be in conflict with previous studies [30]. With our method, we were able to identify and quantify accurately all positive samples compared with other more laborious and expensive methods [16–18]. Although we first used mtDNA from blood samples, this technology can easily be applied to any tissues (Figure 3). Using the optimized conditions, we could detect the heteroplasmy of mt.3243A>G in every tissue tested here from both children and adults.

Previously, the most common method for detection of mt.3243A>G mutation in blood leukocytes is RFLP assay. This method shows only a very faint band with mt.3243A>G mutation loads of over 5 to 10%, below which they are undetectable. Highly sensitive but radioactive assay can detect levels of heteroplasmy down to 1% [18, 30]. The sensitivity of Southern blotting or dot-blot hybridization has been reported to be 2% [19]. Denaturing high-performance liquid chromatography has a limit of 3 to 10% heteroplasmy detection, compared with 5% heteroplasmy discrimination of mt.3243A>G by pyrosequencing [16]. Other new, highly sensitive methods for mt.3243A>G heteroplasmy include ARMS, peptide nucleic acid binding assays, and ligation-mediated PCR, which are both expensive and time-consuming [21–23]. In contrast, our method is relatively simple, rapid, economical, accurate, and safe to quantify the heteroplasmy of mt.3243A>G mutation.

The heteroplasmy load of mt.3243A>G mutation in blood samples is well established. However, several studies have found only a weak correlation between mitochondrial mutation load in blood and clinical phenotype [29]. A good correlation between the frequency of typical clinical features and the level of mutant mtDNA is seen in muscles, brain, urinary cells, or hair follicle [8, 12, 29]. In this study, we detected the mt.3243A>G heteroplasmy from urinary sediments, hair follicles, and leukocytes. We found that the level of heteroplasmy in urinary sediments was higher than that in leukocytes for a single person (Figure 3). This finding was consistent with previous results in that affected tissues (e.g., muscles, brain, or urinary cells) show a higher percentage of the specific heteroplasmy [32–34]. In addition, serial measurements have shown that the percentage of the mt.3243A>G mutation in blood decreases as the patient gets older, but the hair follicle will not [35, 36]. Therefore, the hair follicles or urinary sediments might be the most appropriate samples for detecting the heteroplasmy of mt.3243A>G; however, it needs to be optimized or retreated before direct quantification.

In conclusion, we show that 4% heteroplasmy is reliably and quantitatively detected by TaqMan-MGB qPCR assay. By using our method, it is possible to rapidly and accurately screen a large number of less invasive samples for mt.3243A>G mutation, improving the diagnostic sensitivity and offering a potential application in population screening. The heteroplasmy levels can be detected and quantified simultaneously without further processing, allowing more accurate diagnostic testing of the prevalence of the mt.3243A>G mutation and illuminating the association between clinical phenotype and pathogenic mitochondrial mutations among vairous tissues.

## Figures and Tables

**Table 1:** Sequence of primers and probes used in the current study.

Primer/probe	Sequence (5′-3′)	mtDNA region
Forward RFLP primer	CTCCCTGTACGAAAGGACAAGAGAA	3117 to 3141
Reverse RFLP primer	TATGGGGAGGGGGGTTCATAGTA	3557 to 3579
Forward qPCR primer	ATTATACCCACACCCACCCAAGAAC	3198 to 3222
Reverse qPCR primer	ATGGGTACAATGAGGAGTAGGAGGT	3320 to 3344
Wild-type probe	VIC-CCGGGC**T**CTGCCAT-MGB	3236 to 3249
Mutant probe	6-FAM-CCGGGC**C**CTGCCAT-MGB	3236 to 3249

**Table 2:** Heteroplasmy levels of mt.3243A>G mutation of known negative samples.

Samples	VIC (Ct)	FAM (Ct)	Heteroplasmy levels
1	5.644±0.046	Undetermined	0%
2	5.433±0.046	Undetermined	0%
3	6.024±0.087	Undetermined	0%
4	5.774±0.070	Undetermined	0%
5	7.808±0.130	Undetermined	0%

## Data Availability

The data used to support the findings of this study are included within the article.
